# Quantitative and Chemical Fingerprint Analysis for the Quality Evaluation of Receptaculum Nelumbinis by RP-HPLC Coupled with Hierarchical Clustering Analysis

**DOI:** 10.3390/ijms14011999

**Published:** 2013-01-21

**Authors:** Yan-Bin Wu, Li-Jun Zheng, Jun Yi, Jian-Guo Wu, Ti-Qiang Chen, Jin-Zhong Wu

**Affiliations:** 1 Academy of Integrative Medicine, Fujian University of Traditional Chinese Medicine, Fuzhou 350122, China; E-Mails: wxsq1@163.com (Y.-B.W.); zhenglijun1985@163.com (L.-J.Z.); wjg1419@126.com (J.-G.W.); 2 Department of Science, Fujian Institute of Education, Fuzhou 350001, China; E-Mail: yijun1965@126.com; 3 Fujian Academy of Agricultural Sciences, Fuzhou 350013, China; E-Mail: chen_tiqiang@189.cn

**Keywords:** Receptaculum Nelumbinis, flavonoids compounds, fingerprint analysis, chemometric analysis, quality control

## Abstract

A simple and reliable method of high-performance liquid chromatography with photodiode array detection (HPLC-DAD) was developed to evaluate the quality of Receptaculum Nelumbinis (dried receptacle of *Nelumbo nucifera*) through establishing chromatographic fingerprint and simultaneous determination of five flavonol glycosides, including hyperoside, isoquercitrin, quercetin-3-*O*-β-d-glucuronide, isorhamnetin-3-*O*-β-d-galactoside and syringetin-3-*O*-β-d-glucoside. In quantitative analysis, the five components showed good regression (*R* > 0.9998) within linear ranges, and their recoveries were in the range of 98.31%–100.32%. In the chromatographic fingerprint, twelve peaks were selected as the characteristic peaks to assess the similarities of different samples collected from different origins in China according to the State Food and Drug Administration (SFDA) requirements. Furthermore, hierarchical cluster analysis (HCA) was also applied to evaluate the variation of chemical components among different sources of Receptaculum Nelumbinis in China. This study indicated that the combination of quantitative and chromatographic fingerprint analysis can be readily utilized as a quality control method for Receptaculum Nelumbinis and its related traditional Chinese medicinal preparations.

## 1. Introduction

Lotus (*Nelumbo nucifera* Gaertn.), belonging to the family Nymphaeaceae, is a kind of perennial aquatic herbage plant, which is one of the most important aquatic vegetables widely grown in China, due to its pleasant flavor and high nutritional value, especially its seeds, rhizomes and leaves. It’s easy to be cultivated and distributed in wetlands throughout temperate and tropical Asia from Iran to Japan and from China to Queensland [1]. Up to the year 2002, a total of 572 lotus accessions (including landraces, cultivars and breeding lines) with different germplasm resources were conserved in the National Garden of Aquatic Vegetable in Wuhan, Hubei province, China, including those collections from 153 counties in 18 provinces and lines bred by breeders. According to different purposes or morphological differences, the lotus is usually classified into three types: rhizome lotus, seed lotus and flower lotus. Rhizome lotus is mainly cultivated in Hubei, Jiangsu, Anhui and Zhejiang provinces, seed lotus in Jiangxi, Fujian and Hunan and flower lotus in Wuhan city, Hubei province and Beijing [2].

The previously study reported that different types of lotus have show dissimilar characteristics, which is indicative of their distinct genetic differentiations [3]. There are inextricable links between medicinal plants and their ecological environment in the process of long-term survival competition and natural selection. The genetic variation of active ingredients in germplasm resources is an important factor affecting yield and quality of drugs. To some extent, the formulation of “authentic ingredients” with excellent efficacy is attributed to the action of the “local variety”. In addition, herbs collected at different times and planted in different regions may affect the quality of their chemical composition and the amounts of major bioactive constituents [4].

Receptaculum Nelumbinis, commonly used traditional Chinese medicine (TCM), called Lianfang in Chinese, is derived from the dried receptacle of *N. nucifera*. It is used as an antihemorrhagic agent, especially for excess menstrual bleeding and irregular genital bleeding and also as a remedy for dehydration caused by diarrhea in summer and for prevention of miscarriage in traditional Chinese medicine [5]. In previous bioactivity research on this herb, Receptaculum Nelumbinis have exhibited a wide spectrum of biopharmacological effects, including antioxidation, improving learning and memory abilities, protective effects against experimental myocardial injury and ischemia, radioprotective activity and anti-tumor effects [6–10]. Our previous phytochemical investigations of Receptaculum Nelumbinis have revealed that its main components are phenolic constituents [11]. In the official Chinese pharmacopoeia (China Pharmacopoeia Committee, 2010) [12], only microscopic identification methods were used to identify this medicinal material, and the content determination of marker compounds were not recorded [12]. In the aspects of literature, only the assay of hyperoside and quercetin in Receptaculum Nelumbinis was reported [13,14]. This can’t fully account for all the activities of Receptaculum Nelumbinis and does not meet the need to effectively control the quality for Receptaculum Nelumbinis.

Therefore, in order to control the quality and to clarify the differentiation of chemical constituents in Receptaculum Nelumbinis, a HPLC-DAD method of multiple compounds determination in combination with chemical fingerprinting methodology was developed for the quality evaluation of Receptaculum Nelumbinis. In consideration of the complexity of herb medicine, the HPLC chromatograms are complex multivariate data sets, so minor differences between very similar chromatograms might be missed; the chemical pattern recognition methods, such as similarity analysis (SA) and hierarchical clustering analysis (HCA), were used to reasonably define the class of the herbal medicine and to efficiently evaluate the differentiation of the Receptaculum Nelumbinis samples. We expected that this HPLC method would be helpful for the quality control of Receptaculum Nelumbinis in the future.

## 2. Results and Discussion

### 2.1. Optimization of HPLC Conditions

In order to obtain the chromatograms with better separation of adjacent peaks within a short time, the column, mobile phase and detection wavelength were investigated. Different HPLC columns were tested for better resolution, and then baseline separation of the five constituents was obtained on an Agilent HC-C_18_ column. Acetonitrile-Water system was used as the mobile phase. It could give rise to more peaks, but separation was not satisfactory. According to the literature, acid could achieve better separation for dihydrochalcones [15], thus, 0.2% acetic acid was added to the acetonitrile-water system to further improve the peak shape. Due to a full-scan experiment of the five active components from 200 to 400 nm, 360 nm was selected as the detection wavelength, so that more characteristic peaks could be obtained, and the baseline was well improved on the chromatographic profiles.

### 2.2. Method Validation of Quantitative Analysis

The method was validated in terms of linearity, precision, repeatability, stability and recovery test. The Receptaculum Nelumbinis for method validation was collected from Fujian, China, and the variety of *Nelumbo nucifera* Gaertn was named Taikong 36.

The calibration curve was generated to confirm the linear relationship between the peak area and the concentrations of each reference compound in the test samples. The five standards of hyperoside, isoquercitrin, quercetin-3-*O*-β-d-glucuronide, isorhamnetin-3-*O*-β-d-galactoside and syringetin-3-*O*-β-d-glucoside were accurately weighed, dissolved and diluted with 50% methanol in a volumetric flask to obtain standard solutions for the calibration curves. Calibration curves were peak area *versus* concentration for each analyte. The linear regression equations, correlation coefficients and ranges of calibration curves for the listed flavonoid derivatives are shown in Table 1. The calibration curves showed good linear regression, with correlation coefficience over 0.9998 within test ranges.

A sample of the medicinal material was prepared as described above and was subjected to HPLC analysis six times in the same day to evaluate the precision. The repeatability was examined by the injection of six different samples, which were prepared with the same sample preparation procedure. Variations were expressed as relative standard deviations (RSD). Table 2 showed the results of the tests of precision and repeatability. The stability was analyzed in 0, 4, 8, 12, 24 and 48 h within 2 days. Stability was expressed as the RSD, and the values were less than 0.69% for the five compounds (Table 2). The recovery test was determined by the standard addition method. Five flavonol glycosides were added to the samples, and then, the extraction and analysis were performed according to the above sample preparation procedure. The mean recovery was calculated according to the following formula: recovery (%) = [(found amount − original amount)/spiked amount] × 100% and RSD (%) = (SD/mean) × 100%. The mean recovery of the five flavonoids compounds was 98.31%–100.32%, and their RSD values were less than 3.00% (Table 2).

### 2.3. Establishment of Chromatographic Fingerprint of Receptaculum Nelumbinis and Similarity Analysis (SA)

**Figure 1:**
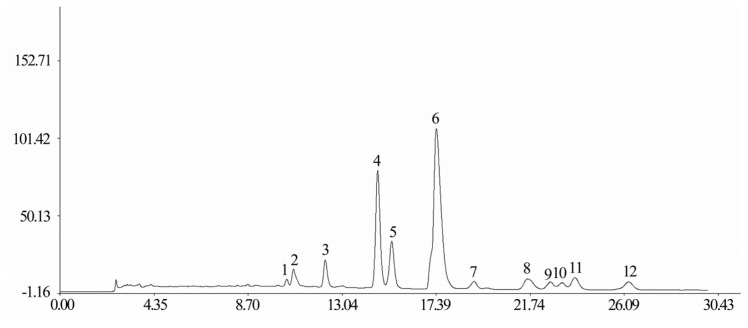
Average artificial HPLC fingerprint common pattern of Receptaculum Nelumbinis based on 20 samples.

**Figure 2:**
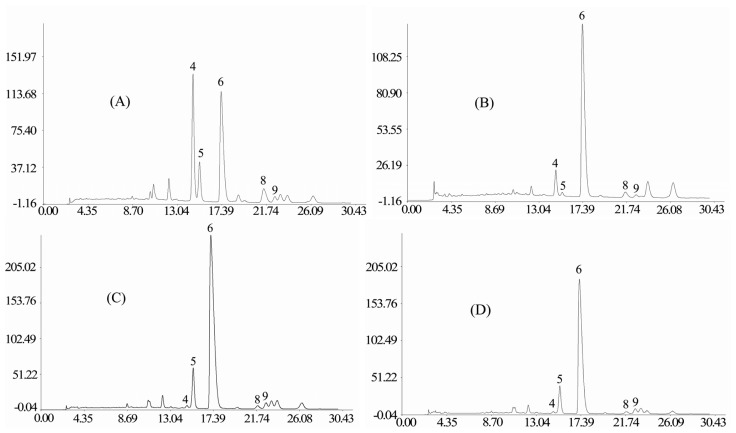
The representative standard fingerprints of Receptaculum Nelumbinis obtained by Similarity Evaluation System: (**A**) L1; (**B**) L11; (**C**) L13; (**D**) L17.

To standardize the HPLC profile, 20 samples of Receptaculum Nelumbinis were analyzed, and all chromatograms were introduced into the Computer-Aided Similarity Evaluation System for Chromatographic Fingerprint of TCM (China Committee of Pharmacopeia, 2004). Peaks that existed in all chromatograms of samples with reasonable heights and good resolutions were assigned as “common peak” for Receptaculum Nelumbinis. As shown in Figure 1, there are 12 distinct common peaks (from peak 1 to peak 12) in the HPLC fingerprint common patterns from the 20 samples of Receptaculum Nelumbinis, and the representative standard fingerprints of the investigated samples is shown in Figure 2. Five common peaks (peak 4, 5, 6, 8 and 9) were identified as hyperoside, isoquercitrin, quercetin-3-*O*-β-d-glucuronide, isorhamnetin-3-*O*-β-d-galactoside and syringetin-3-*O*-β-d-glucoside.

The similarities of chromatograms for the varieties of the receptacles of *N. nucifera* were calculated using the similarity evaluation system recommended by SFDA. Similarity comparison of the standard fingerprints of different samples showed that the similarity ranged from 0.282 to 0.966 (Table 3). The results (Table 3) showed that sample L11, L13 and L17 have a small similarity with its similarity, respectively, as 0.867, 0.283 and 0.282. The similarity values of the other samples were more than 0.918. These results indicated that the chemical composition and content in the Receptaculum Nelumbinis varied significantly.

### 2.4. Quantitative Determination of Five Compounds in Receptaculum Nelumbinis

**Figure 3:**
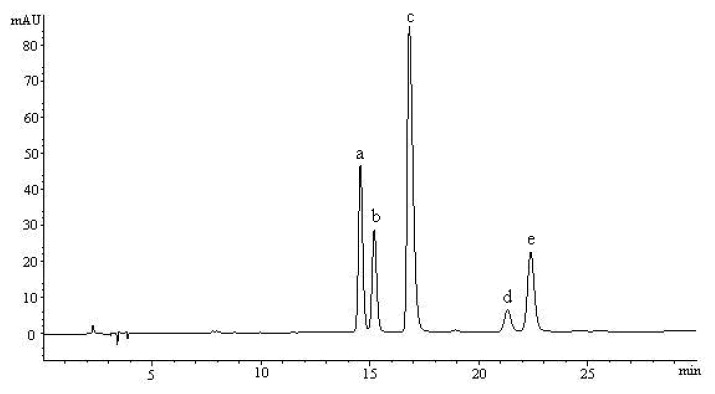
HPLC chromatogram of mixture standard of the five compounds: (**a**) Hyperoside; (**b**) Isoquercitrin; (**c**) Quercetin-3-*O*-β-d-glucuronide; (**d**) Isorhamnetin-3-*O*-β-d-galactoside; (**e**) Syringetin-3-*O*-β-d-glucoside.

According to the contents and pharmacological properties of major constituents in Receptaculum Nelumbinis, the peak of hyperoside, isoquercitrin, quercetin-3-*O*-β-d-glucuronide, isorhamnetin-3-*O*-β-d-galactoside and syringetin-3-*O*-β-d-glucoside were chosen as reference peaks. The contents of the five compounds in the twenty varieties of the receptacles of *N. nucifera* from different sources in China were determined by the establishing HPLC method. The representative HPLC chromatogram of the five compounds is shown in Figure 3. Each sample was analyzed in triplicate to determine the mean content (mg/g), and the results are listed in Table 4. The quantitative analysis results showed that the twenty varieties of the receptacles of *N. nucifera* generally contained the five flavonol glycosides, and the content of the five compounds were significantly different. The content ranges for hyperoside, isoquercitrin, quercetin-3-*O*-β-d-glucuronide, isorhamnetin-3-*O*-β-d-galactoside and syringetin-3-*O*-β-d-glucoside were 0.1–9.1, 0.1–6.0, 9.3–72.2, 0.3–3.3 and 0.1–1.9 mg/mL, respectively. The results in Table 4 showed that the content of each flavonol glycoside in different samples were significantly different, which is consistent with that of HPLC fingerprint analysis. Moreover, hyperoside, isoquercitrin and quercetin-3-*O*-β-d-glucuronide were found to be predominant among the five determined analytes. Many studies have shown that hyperoside, isoquercitrin and quercetin-3-*O*-β-d-glucuronide have exhibited a wide spectrum of biopharmacological effects, including beneficial cardiovascular effect, antioxidation, anti-hypertrophic effect on vascular smooth muscle cell, antiviral, anti-inflammatory and anti-tumor effect [11,16–20]. The high yield of hyperoside, isoquercitrin and quercetin-3-*O*-β-d-glucuronide in 50% ethanol extract may contribute to the curative effect of Receptaculum Nelumbinis.

According to the quantitative analysis results, it was suggested that the genetic variation was one of the key factors affecting the contents of bioactive constituents. The results also indicated that the internal qualities of 20 batches of Receptaculum Nelumbinis samples from different sources with different varieties had marked variations, and the quality control needed evaluation by chemical fingerprinting. Multiple factors for Receptaculum Nelumbinis, such as various regions, source and different harvesting time various, would accordingly result in the differences in their qualities. Thus, the selection of the stable source of the Receptaculum Nelumbinis is quite important and meaningful for the clinical effect and quality evaluation of this medicine.

### 2.5. Hierarchical Clustering Analysis (HCA)

HCA is a multivariate analysis technique that is used to sort samples into groups [21]. The HCA method is well known and has been applied for fingerprint analysis, because it is a nonparametric data interpretation method and simple to use [21–24]. HCA provides a visual representation of complex data. A method called average linkage between groups was applied, and Pearson correlation was selected as a measurement. The method can classify different herbs by measuring the peak areas from their corresponding LC fingerprints. The common characteristic peaks, which were calculated by the Similarity Evaluation System, were selected for the hierarchical cluster analysis [4].

**Figure 4:**
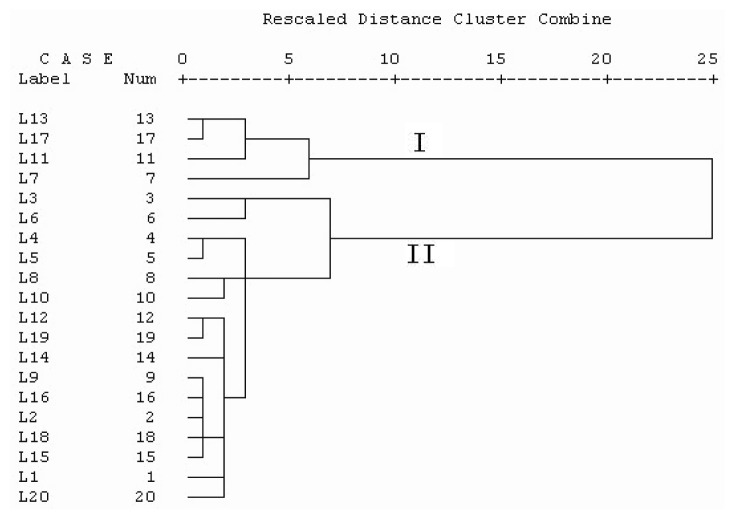
Results of hierarchical cluster analysis of 20 Receptaculum Nelumbinis samples (dendrogram using average linkage between groups).

In order to assess the resemblance and differences of these samples, a hierarchical agglomerative clustering analysis of Receptaculum Nelumbinis samples was performed based on the relative peak areas of all the 12 characteristics chromatographic peaks. The peak areas of characteristics constituents in 20 batches of Receptaculum Nelumbinis samples from various sources formed a matrix of 12 × 20. The results of HCA were shown in Figure 4 from which the quality characteristics were revealed more clearly. The results of the hierarchical cluster analysis showed that the samples could be divided into two quality clusters. Among them, Cluster I includes the samples L7, L11, L13 and L17 and the other in Cluster II. Cluster I was distinguished as hyperoside—poor chemotype—which contains less hyperoside than the Cluster II. These results were in correspondence to the SA. The low concentration of hyperoside in the Cluster I may be due to the poor herb quality of Receptaculum Nelumbinis. This indicated that hyperoside could be used as a marker compound to distinguish the Receptaculum Nelumbinis with different quality. The results of HCA could be validated each other and provided more references for the quality evaluation of Receptaculum Nelumbinis.

## 3. Experimental Section

### 3.1. Plant Materials and Reagents

Twenty Receptaculum Nelumbinis populations were collected from different regions of China, and all voucher specimens were taxonomically identified based on morphological characteristics by Professor J. Z. Wu and deposited at Herbarium of Academy of Integrative Medicine, Fujian University of Traditional Chinese Medicine in Fuzhou 350108, China.

HPLC grade acetonitrile and methanol were purchased from Fisher Scientific (Pittsburgh, PA, USA). HPLC grade water was prepared using a Milli-Q water purification system (Millipore, Bedford, MA, USA). Analytical grade methanol, ethanol and acetic acid were purchased from Sinopharm Chemical Reagent Co. Ltd, Shanghai, China. All the solutions were filtered through 0.45 μm membranes (Schleicher & Schuell, Dassel, Germany) and degassed by ultrasonic bath before use.

### 3.2. Instrument and Chromatographic Conditions

The chromatographic separation was performed on an Angilent 1200 HPLC system (Agilent Technologies Inc., Santa Clara, CA, USA), equipped with a quaternary pump, an autosampler, a degasser, an automatic thermostatic column compartment, a DAD detector and a computer with a Chemstation software program for analysis of the HPLC data. Agilent HC-C_18_ reversed-phase column (250 mm × 4.6, 5 μm) together with an Agilent HC-C_18_ guard column (12.5 mm × 4.6, 5 μm) were used with column temperature set at 25 °C. HPLC-DAD detection was used for purity assay of reference compounds. The mobile phase consisted of acetonitrile (A) and 0.2% acetic acid (*v*/*v*, B) using a gradient program of 16%–19.6% A in 0–6 min, 19.6%–20.7% A in 6–30 min. This was followed by a 10 min equilibration period to the injection of each sample. The flow rate was 1 mL/min, and detection wavelength was set at 360 nm; an aliquot of 10 μL solution was injected for acquiring chromatograms.

### 3.3. Preparation of Standard Solutions

Hyperoside, isoquercitrin, quercetin-3-*O*-β-d-glucuronide, isorhamnetin-3-*O*-β-d-galactoside and syringetin-3-*O*-β-d-glucoside were extracted, isolated and purified from Receptaculum Nelumbinis in our laboratory. The chromatogram of the five mixture compounds was shown in Figure 4. All were identified using ESI-MS, ^1^H-NMR and ^13^C-NMR spectrometric techniques. The purity of each compound was determined to be higher than 98% by HPLC. Their structures and the detailed procedures for isolation and spectrometric identification have been reported in another paper [11].

Five reference compounds were accurately weighed and dissolved in 20% acetonitrile, then diluted to appropriate concentration ranges for the establishment of calibration curves. All stock and working standard solutions were stored at 4 °C until used for analysis.

### 3.4. Preparation of Sample Solutions

The open-air dried lotus receptacles were cut into small pieces and ground into powder, and then 1.0 g of sample fine powder was extracted twice with 25 mL of 50% ethanol by ultrasonic for 30 min. The extracts was filtered and evaporated under vacuum, the residues were dissolved with 10 mL 20% acetonitrile solution and sonicated for 10 min. The sample solution was filtered through a 0.45 μm membrane filter prior to HPLC analysis, and the injection volume was 10 μL.

### 3.5. Data Analysis

The chromatographic profiles of all extracts were performed by professional software named Similarity Evaluation System for Chromatographic Fingerprint of Traditional Chinese Medicine (Version 2004 A), which was recommended by the State Food and Drug Administration of China (SFDA) for evaluating similarities of chromatographic profiles of TCM [19]. The hierarchical cluster analysis (HCA) of samples was performed using SPSS software (SPSS 16.0 for Windows Vista™, SPSS Inc., Chicago, IL, USA).

## 4. Conclusions

In this paper, a HPLC fingerprint and quantitative analysis method was developed to evaluate the quality of Receptaculum Nelumbinis from different sources. The method was well validated by systematically comparing chromatograms of all samples from different sources and certified helpfully to improve the quality control. Meanwhile, the chemometrics methods were applied with the HPLC fingerprint techniques for analysis of chemical variation of Receptaculum Nelumbinis samples. Chemometrics analysis indicated that the quality of Receptaculum Nelumbinis have no significant relativity with geographic location and germplasm resources. HCA could distinguish these samples as different chemical-types, but not different geographic population and germplasm resources. In the view of results of content analysis, the samples L7, L11, L13 and L17 have a lower content of hyperoside, with its content, respectively, as 0.8, 1.1, 0.1 and 0.2 mg/g. This also explained why samples L7, L11, L13 and L17 were grouped as the same type in HCA analysis and have a small similarity. Furthermore, five marker constituents were found to be specific variables, which could provide the most discrimination and quality control of Receptaculum Nelumbinis by quantitative analysis. The results demonstrated that the chemometrics techniques, such as SA and HCA, were able to classify samples objectively and successfully in accordance with their chemical constituents and content. Further, the method is a powerful, practical tool for quality control of Receptaculum Nelumbinis samples or other related traditional Chinese medicinal preparations.

## Figures and Tables

**Table 1:** Regression equation and correlation coefficient of calibration curves for the five compounds.

Compound	Regression equation^a^	*R*^b^	Linearity range (μg/mL)
Hyperoside	*Y* = 25.303*x* + 13.352	0.9998	8–104 μg/mL
Isoquercitrin	*Y* = 16.058*x* + 22.746	0.9991	2–64 μg/mL
Quercetin-3-*O*-β-d-glucuronide	*Y* = 9.6542*x* − 26.718	0.9998	30–960 μg/mL
Isorhamnetin-3-*O*-β-d-galactoside	*Y* = 19.097*x* − 1.1527	0.9999	1.4–42.6 μg/mL
Syringetin-3-*O*-β-d-glucoside	*Y* = 13.305*x* + 0.9321	0.9997	0.7–22.8 μg/mL

a *Y* peak area, *x* concentration of compound (μg/mL);

b *R* = correlation coefficient, *n* = 6.

**Table 2:** Precision, reproducibility, stability and recovery of the five compounds.

Compound	Precision RSD (%) (*n* = 6)	Reproducibility RSD (%) (*n* = 6)	Stability RSD (%) (*n* = 6)	Recovery (%) (*n* = 6) Mean ± RSD (%)
Hyperoside	0.07	1.99	0.09	99.23 ± 2.61
Isoquercitrin	0.14	2.37	0.17	99.72 ± 2.84
Quercetin-3-*O*-β-d-glucuronide	0.48	2.88	0.68	99.54 ± 2.91
Isorhamnetin-3-*O*-β-d-galactoside	0.08	2.44	0.18	98.31 ± 3.00
Syringetin-3-*O*-β-d-glucoside	0.49	2.96	0.69	100.32 ± 2.71

**Table 3:** The similarities of the chromatograms of twenty varieties of the receptacles of *N. nucifera*.

Samples	Similarities
L1	0.956
L2	0.962
L3	0.934
L4	0.957
L5	0.954
L6	0.941
L7	0.918
L8	0.959
L9	0.966
L10	0.959
L11	0.867
L12	0.961
L13	0.283
L14	0.959
L15	0.965
L16	0.966
L17	0.282
L18	0.965
L19	0.956
L20	0.956

**Table 4:** Contents (mg/g) of five compounds in twenty varieties of the receptacles of *N. nucifera* (*n* = 3).

Samples	Cultivated varieties	Collected location	Collection time	Contents (mg/g)				

				1^a^	2	3	4	5
L1	Taikong 36	Jianou, Fujian	October 2010	7.4	3.9	27.0	2.1	1.0
L2	Zajiao 8236	Jianou, Fujian	October 2010	6.2	2.9	29.1	1.8	0.9
L3	Jianjibaihualian	Jianou, Fujian	October 2010	5.5	4.9	18.4	1.3	0.6
L4	Guangchang Taikong 3	Guangchang, Jiangxi	September 2010	2.5	1.4	14.7	1.2	0.7
L5	Baihualian	Guangchang, Jiangxi	September 2010	1.4	0.5	8.5	0.6	0.3
L6	Shilihe 1	Hangzhou, Zhejiang	September 2010	2.9	1.8	9.3	0.3	0.4
L7	Liyebailian	Hangzhou, Zhejiang	September 2010	0.8	0.2	9.4	0.3	0.1
L8	Jianxuan 17	Hangzhou, Zhejiang	August 2010	6.8	6.0	41.8	1.4	1.5
L9	Taikong 3	Hangzhou, Zhejiang	August 2010	7.9	3.8	33.7	1.9	1.1
L10	Ganxuan 62	Hangzhou, Zhejiang	August 2010	2.6	1.7	14.4	0.4	0.4
L11	Jinfunong	Hangzhou, Zhejiang	August 2010	1.1	0.1	30.6	0.6	0.4
L12	Dahonglian	Hangzhou, Zhejiang	August 2010	7.3	3.6	29.7	0.9	1.0
L13	Guanshanglian	Yuanmingyuan, Beijing	August 2010	0.2	5.5	76.2	0.6	1.4
L14	Baoyingmeirenhonglian	Baoying, Jiangsu	August 2010	4.4	3.2	19.3	0.8	0.5
L15	Honghelian	Honghu, Hubei	August 2010	4.1	2.1	21.3	1.2	0.8
L16	Jianlian	Jianning, Fujian	August 2010	4.1	2.1	21.3	1.2	0.8
L17	Baiyangdingyeshenglian	Baoding, Hebei	August 2010	0.1	3.6	49.2	0.4	1.1
L18	Xingkongmudan	Guangchang, Jiangxi	August 2010	7.5	4.2	34.8	1.5	0.9
L19	Cunsanlian	Xiangtan, Hunan	August 2010	4.8	2.8	19.8	0.6	0.7
L20	Taikong 1	Hangzhou, Zhejiang	August 2010	9.1	3.2	36.5	3.3	1.9

a The data was present at average of duplicates. 1: Hyperoside; 2: Isoquercitrin; 3: Quercetin-3-*O*-β-d-glucuronide; 4: Isorhamnetin-3-*O*-β-d-galactoside; 5: Syringetin-3-*O*-β-d-glucoside.
